# Effects of dietary lysine restriction on inflammatory responses in piglets

**DOI:** 10.1038/s41598-018-20689-3

**Published:** 2018-02-05

**Authors:** Hui Han, Jie Yin, Bin Wang, Xingguo Huang, Jiming Yao, Jie Zheng, Wenjun Fan, Tiejun Li, Yulong Yin

**Affiliations:** 1 Key Laboratory of Agro-ecological Processes in Subtropical Region, Institute of Subtropical Agriculture, Chinese Academy of Sciences; Scientific Observing and Experimental Station of Animal Nutrition and Feed Science in South-Central, Ministry of Agriculture; Hunan Provincial Engineering Research Center for Healthy Livestock and Poultry Production, Changsha, Hunan 410125 P.R. China; 2 National Engineering Laboratory for Pollution Control and Waste Utilization in Livestock and Poultry Production, Hunan, 410125 P.R. China; 3 University of Chinese Academy of Sciences, Beijing, 100039 P.R. China; 4 School of Food, Jiangsu Food & Pharmaceutical Science College Higher Education Park in Huaian, Huaian, Jiangsu 223005 P.R. China; 5 Department of Animal Science, Hunan Agriculture University, Changsha, Hunan 410128 P.R. China; 6 Guangdong Wangda Group Academician Workstation for Clean Feed Technology Research and Development in Swine, Guangdong Wangda Group Co., Ltd., Guangzhou, Guangdong, 510663 P.R. China; 7 Hunan Co-Innovation Center of Animal Production Safety, CICAPS, Changsha, Hunan 410128 P.R. China

## Abstract

The aim of this study was to investigate the effects of lysine restriction on inflammatory responses in piglets. 38 male piglets with similar body weight of 9.62 kg were randomly divided into control group (basal diet) and lysine-restricted group (diet containing 70% lysine of the control diet). The results showed that lysine restriction increased the serum concentration of IgG an IgM. Piglets fed the lysine-restricted diet exhibited overexpression of interleukin-8 (IL-8) in the kidney (P < 0.05) and IL-6 and IL-4 in the spleen (P < 0.05). The mRNA abundances of IL-4 in the kidney (P < 0.05) and IL-10 in the liver (P < 0.05) were significantly lower in the lysine-restricted group compared with the control group. Meanwhile, lysine restriction increased the mRNA level of Tlr8 in the kidney (P < 0.05) but decreased the mRNA level of Tlr8 in the liver (P < 0.05). Finally, lysine restriction markedly enhanced extracellular signal regulated kinases 1/2 (ERK1/2) phosphorylation in the kidney and liver and nuclear transcription factor kappa B (NF-κB) was activated in the liver and spleen in response to dietary lysine restriction. In conclusion, lysine restriction affected inflammatory responses in the kidney, liver, and spleen via mediating serum antibody volume, inflammatory cytokines, Tlrs system, and ERK1/2 and NF-κB signals in piglets.

## Introduction

Amino acids are critically important for the growth, health, and disease in piglets^1^. Lysine is one of the building blocks for synthesis of proteins, peptides and non-peptide molecules^2^, which are involved in various biochemical and physiological process. In our previous reports, we found that dietary different dosages of lysine influence intestinal morphology and expressions of amino acid transporters, which further mediate intestinal absorption and metabolism of amino acids^3,4^. More recently, lysine deficiency *in vivo* and *in vitro* was investigated in our lab and the results showed that lysine deficiency affects cell cycle arrest, apoptosis, and amino acid metabolism, which may be associated with the mammalian target of rapamycin (mTOR) signal^5^.

Dietary lysine deficiency also impairs both antibody responses and cell-mediated immune responses^1,6,7^. However, the effect of lysine restriction on inflammatory response is still obscure. Thus, the present study aimed to investigate the inflammatory status of the kidney, liver, and spleen in piglets after exposure to a lysine-restricted diet.

## Results

### Lysine restriction increased serum concentration of IgG and IgM

The serum concentration of IgG and IgM were significantly higher (P < 0.01) in piglets from the lysine-restricted group when compared with the control group (Table 1).

**Table 1:** Serum concentration of IgG and IgM in piglets.

Item	100%Lysine	70%Lysine	P
IgG, mg/mL	0.69±0.10	0.97±0.17^a^	<0.01
IgM, mg/mL	0.22±0.03	0.47±0.05^a^	<0.01

### Lysine restriction upregulated pro-inflammatory cytokines

**Figure 1:**
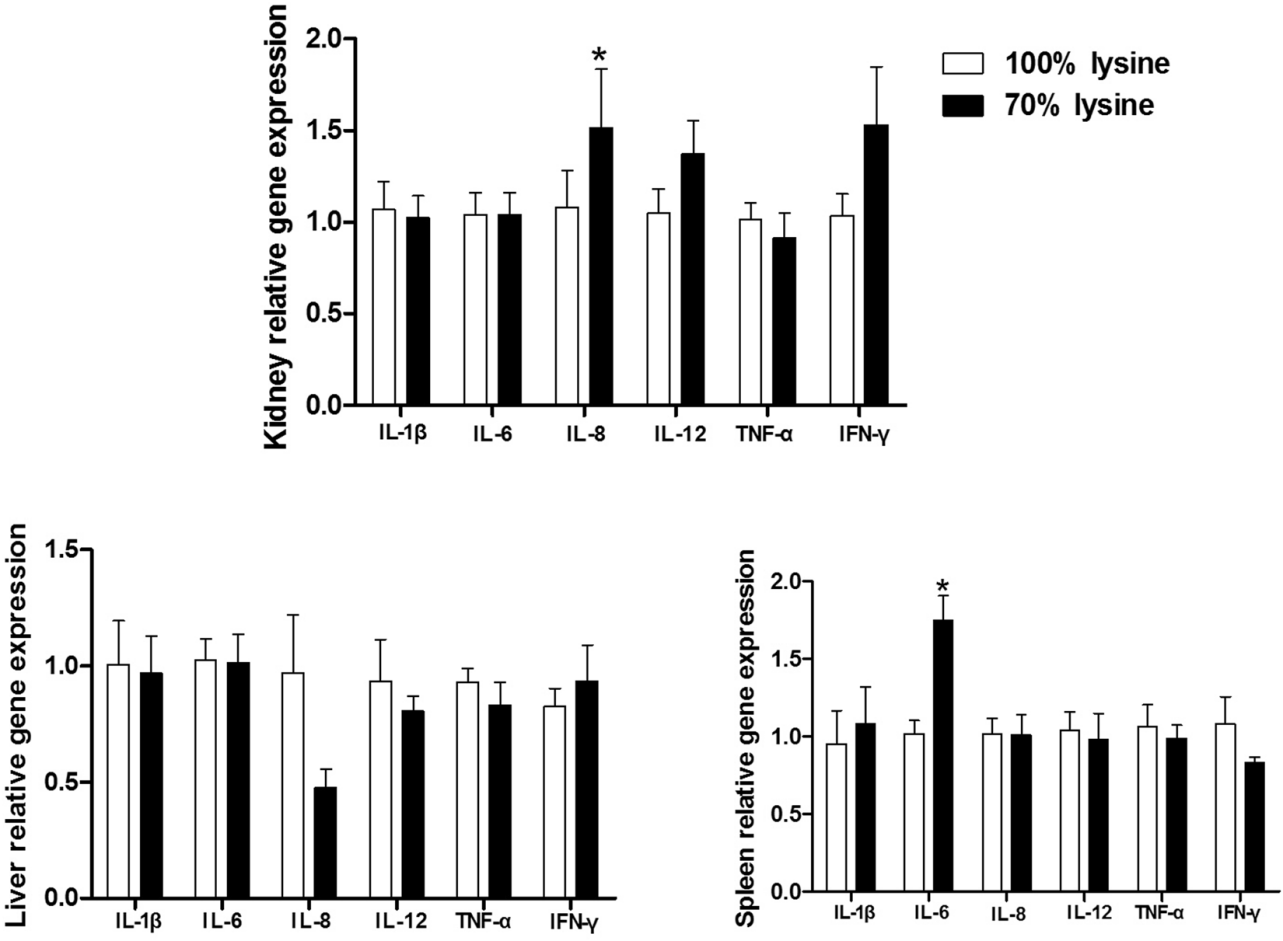
Pro-inflammatory cytokines mRNA levels in piglets fed a basal diet (100% lysine) or a lysine-restricted diet containing 70% lysine of the basal diet. Values are means ± SEMs, n = 7. *Different from control, P < 0.05.

Expressions of pro-inflammatory cytokines (IL-1β, IL-6, IL-8, IL-12, tumor necrosis factor-α (TNF-α), and interferon-gamma (IFN-γ)) were determined in the kidney, liver, and spleen (Fig. 1). The results showed that lysine restriction markedly increased mRNA abundances of IL-8 in the kidney and IL-6 in the spleen (P < 0.05). Meanwhile, IL-12 and IFN-γ expressions in the kidney tended to decrease in lysine-restricted group, while the difference was insignificant (P > 0.05).

### Lysine restriction influenced anti-inflammatory cytokines

**Figure 2:**
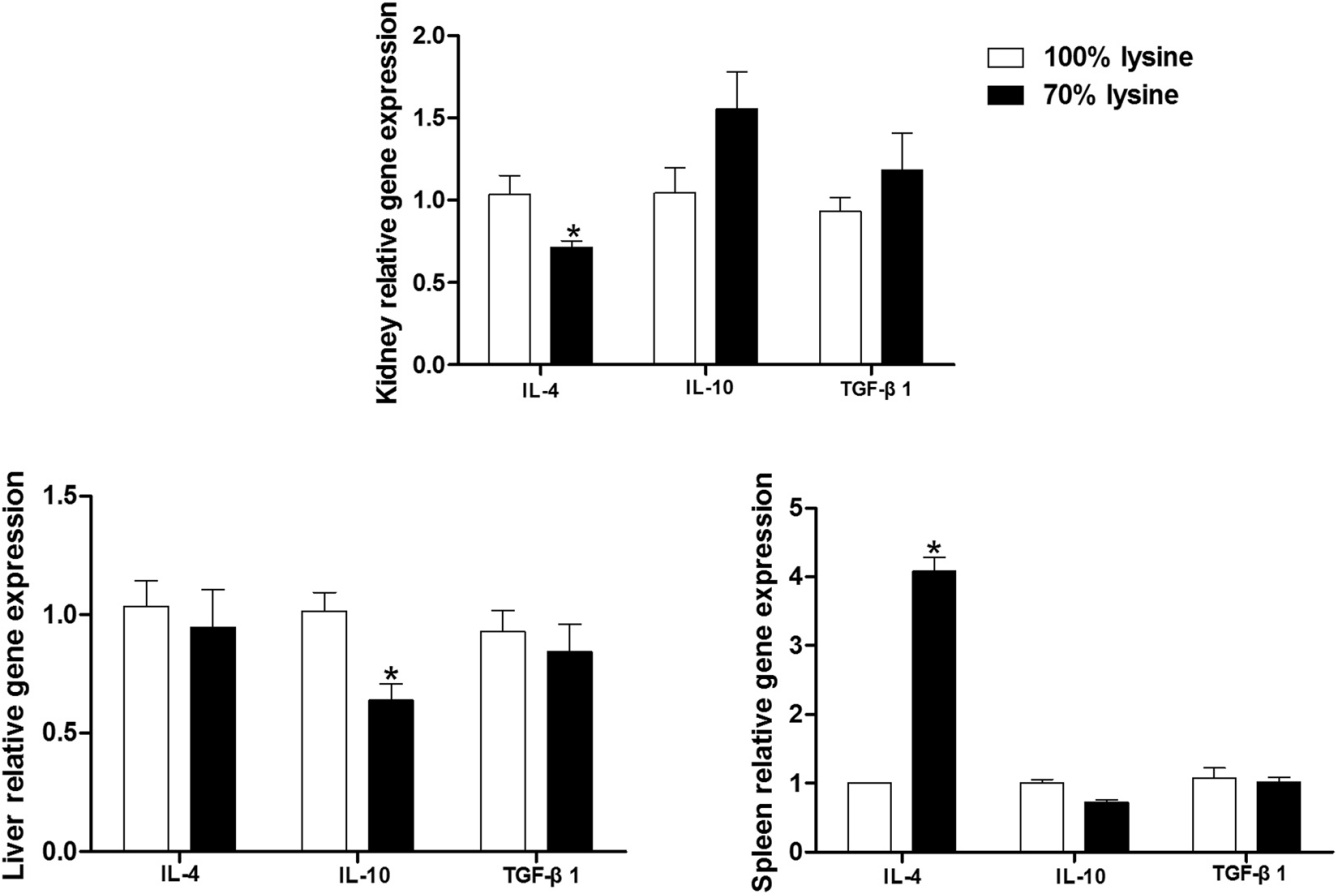
Anti-inflammatory cytokines mRNA levels in piglets fed a basal diet (100% lysine) or a lysine-restricted diet containing 70% lysine of the basal diet. Values are means ± SEMs, n = 7. *Different from control, P < 0.05.

Dietary lysine restriction decreased the mRNA level of IL-4 in the kidney (P < 0.05) and mRNA level of IL-10 in the liver (P < 0.05). While the IL-4 mRNA level in the spleen were markedly higher in the lysine-restricted group compared to the control group (P < 0.05) (Fig. 2).

### Effects of lysine restriction on toll-like receptors (Tlrs) system

**Figure 3:**
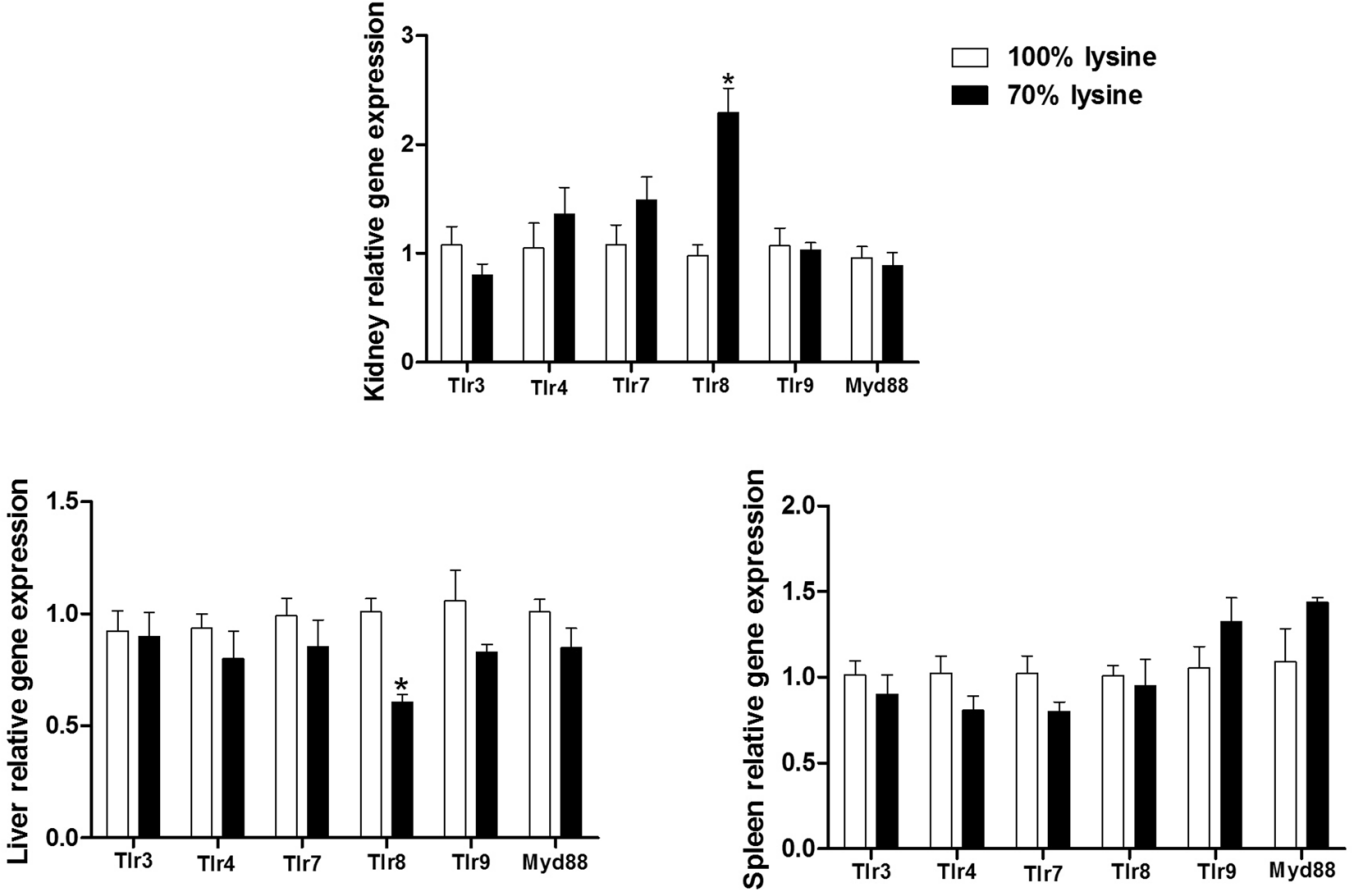
Tlrs mRNA levels in piglets fed a basal diet (100% lysine) or a lysine-restricted diet containing 70% lysine of the basal diet. Values are means ± SEMs, n = 7. *Different from control, P < 0.05.

Tlrs are widely demonstrated to involve in the activation of inflammatory response. Thus, expressions of Tlr3, 4, 7, 8, 9, and Myd88 were determined in the kidney, liver, and spleen (Fig. 3). Lysine restriction increased the mRNA level of Tlr8 in the kidney (P < 0.05) but decreased the mRNA level of Tlr8 in the liver (P < 0.05). Furthermore, lysine restriction exhibited little effect on expression of other Tlrs and myeloid differentiation 88 (Myd88) in the kidney, liver, and spleen.

### Lysine restriction induced the abundance of extracellular signal regulated kinases 1/2 (ERK1/2) and nuclear transcription factor kappa B (NF-κB) proteins

**Figure 4:**
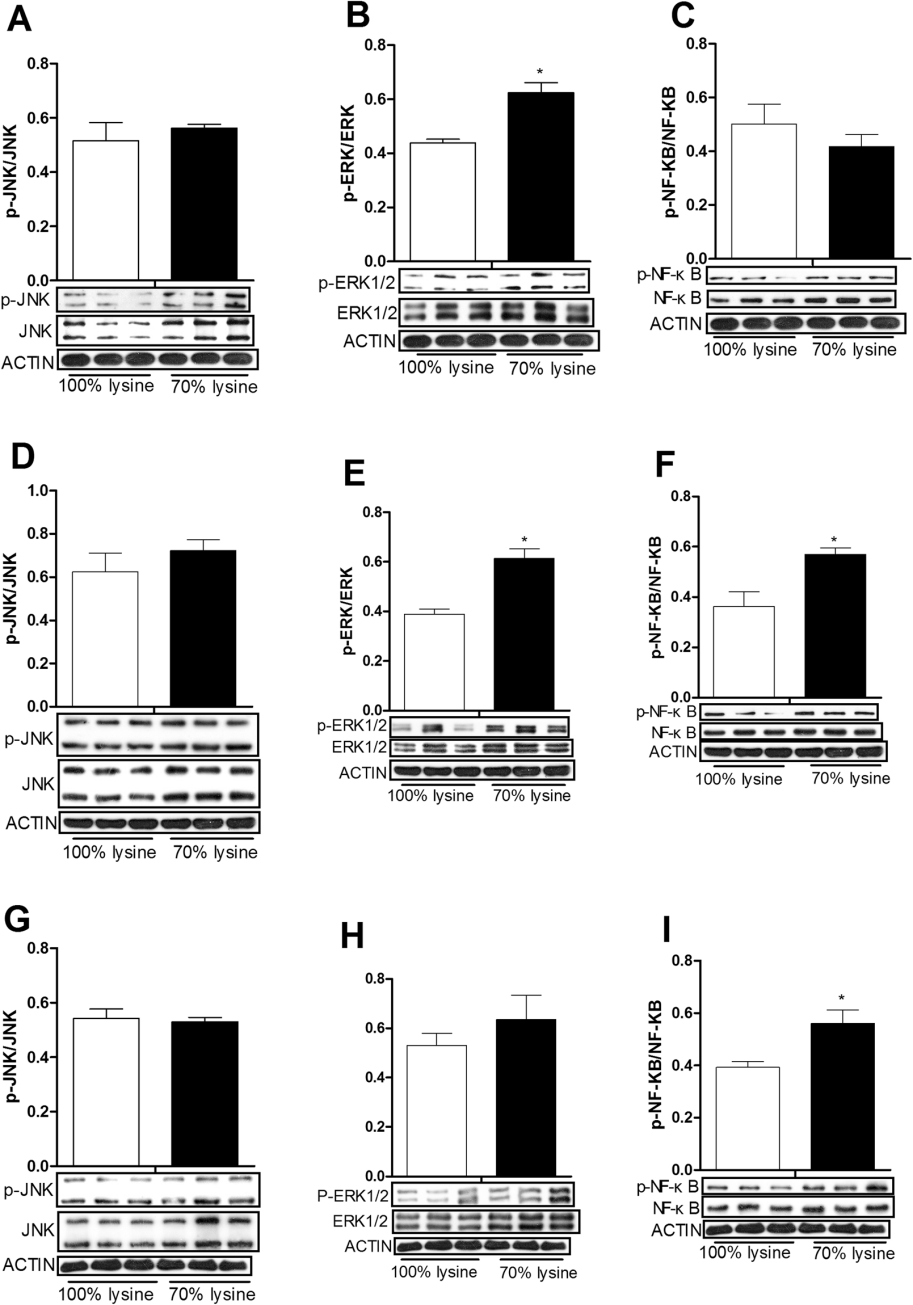
The ration of phosphorylated JNK abundance to total JNK in the kidney (**A**), liver (**D**), and spleen (**G**) of piglets fed a basal diet (100% lysine) or a lysine-restricted diet containing 70% lysine of the basal diet. The ration of phosphorylated ERK1/2 abundance to total ERK1/2 in the kidney (**B**), liver (**E**), and spleen (**H**) of piglets fed a basal diet (100% lysine) or a lysine-restricted diet containing 70% lysine of the basal diet. The ration of phosphorylated NF-κB abundance to NF-κB in the kidney (**C**), liver (**F**), and spleen (**I**) of piglets fed a basal diet (100% lysine) or a lysine-restricted diet containing 70% lysine of the basal diet. Each image shows three samples from 100% Lysine group and three samples from 70% Lysine group. *Different from control, P < 0.01.

ERK1/2 signal was markedly activated in the kidney and liver and NF-κB signal was upregulated in the liver and spleen of lysine restricted piglets evidenced by the enhanced phosphorylation ratio of ERK1/2 and NF-κB (P < 0.01) (Fig. 4).

## Discussion

Lysine is the first limiting amino acid for piglets and is one of the building blocks for the synthesis of proteins^8^. For this reason, inadequate lysine intake can limit the synthesis of inflammatory-related proteins (including cytokines)^9^. Numerous studies have demonstrated that the intake of amino acid affect the inflammatory responses of animals^7,10^. What’s more, it was reported that the deficiency of dietary lysine also impaired animal immune responses^9,11^.

The serum antibody volume has been widely used to evaluated the humoral immunity^12^. IgG and IgM, two major serum immunoglobulins, are key components humoral immunity in all mammals^13^ and protect the extravascular compartment against pathogenic virus and microorganisms^9^. In this study, dietary lysine restriction decreased the serum concentration of IgG and IgM. Pro-inflammatory cytokines (including IL-6 and IL-8) serve as an important role in mediating inflammatory and immune responses^14–17^. IL-4 is involved in all major aspects of inflammatory responses^18^. IL-10, an anti-inflammatory cytokine, down-regulates macrophage activity in swine^19,20^. In this study, the mRNA abundance of IL-8 in the kidney and IL-6 in the spleen were significantly higher in the lysine-restricted group compared with the control group. We also found that lysine restriction markedly decreased the abundance of IL-4 in the kidney and IL-10 in the liver, but significantly increased the abundance of IL-4 in the spleen. Tlrs are a family of pathogen recognition receptors which promote innate immunity^14^. Tlrs activate the expression of pro-inflammatory, such as IL-6 and TNF-α^21^. Myd88 plays an important role in the Tlr signaling pathway^22^. Dietary arginine supplementation has effects on the activation of Tlrs^23^. In the present study, lysine restriction influenced the expression of Tlr8 in the kidney and liver of piglets. These results showed that lysine restriction affect inflammatory response via mediating serum antibody volume, inflammatory cytokines, and Tlrs. Notably, the current results showed a tissue-dependent of gene expressions, which might be caused by different functions of these tissues. For example, liver mainly contributes to metabolism and kidney involves in excretion and re-absorption. Similarly, we also noticed that expressions of Tlr system varied from different sections of intestine (duodenum, jejunum, and ileum)^24^.

NF-κB pathway plays an important role in inflammation by mediating synthesis of pro-inflammatory (i.e. IL-6 and IL-8)^25^. Mitogen-activated protein kinase (MAPK) pathway involves in nuclear translocation of NF-κB and contributes to the production of inflammatory cytokines^26,27^. ERKs and c-Jun N-terminal protein kinase (JNK) are members of MAPK family, which is associated with inflammation^28^. Amino acids have been demonstrated to activate NF-κB and MAPK signaling pathways to regulate expression of pro-inflammatory cytokines and inflammation^23^. Similarly, in this study, lysine restriction activated ERK1/2 and NF-κB signals, which might further involve in immune and inflammatory responses. Our previous study has revealed that lysine deficiency induced apoptosis^5^, which is highly associated with inflammatory response^29^. Thus, it is not surprising to uncover that lysine restriction induces inflammatory response.

Taken together, this study indicated that lysine restriction can induce inflammatory via mediating serum concentration of IgG and IgM, the expression of inflammatory cytokines, Tlrs, and ERK1/2 and NF-κB signals in the kidney, liver, and spleen of piglets.

## Materials and Methods

### Animals and Experimental Design

This study was conducted in accordance with the guidelines of the Institute of Subtropical Agriculture, Chinese Academy of Sciences. All experimental protocols were approved by animal ethical committee of the Institute of Subtropical Agriculture, Chinese Academy of Sciences. 38 male piglets (about 35-day old, 9.62 ± 0.30 kg) were randomly divided into 2 groups: a control group and a lysine-restricted group. Piglets in the control group were received the basal diet according to the NRC (2012) (Table 2), whereas piglets in the lysine-restricted group were fed a lysine-restricted diet containing 70% lysine of the control group. Piglets were individually housed in cages and had ad libitum access to drinking water and feed for 21 days. Then 7 animals were sampled randomly from each group. Blood samples from the overnight fasting piglets were collected in plastic uncoated tubes. Sera were obtained by centrifugation at 3000 rpm for 20 min and stored at −20 °C until analysis for IgG and IgM. After blood sampling, the piglets were sacrificed for kidney, liver, and spleen collection.

**Table 2:** Ingredient and nutrient composition of the experimental diets.

	Lysine concentration (%)	
	100	70
**Ingredients, %**		
Corn	67.00	67.00
Soybean meal	17.90	17.90
Whey powder	4.30	4.30
Fish meal	3.90	3.90
Soybean oil	2.60	2.60
Lysine	0.65	0.18
Methionine	0.19	0.19
Threonine	0.21	0.21
Tryptophane	0.04	0.04
Alanine	0.47	0.95
CaHCO_3_	0.74	0.74
Limestone	0.70	0.70
Salt	0.30	0.30
Premix^*^	1.00	1.00
**Nutrient concent**		
Digestive energy, MJ/kg diet	14.52	14.52
Crude protein, %	17.18	17.18
Lysine, %	1.23	0.86
Methionine+Cysteine	0.67	0.67
Threonine	0.72	0.72
Tryptophan	0.19	0.19
Calcium, %	0.70	0.70
Total phosphorus, %	0.55	0.55

*Premix provided the following per kilogram of the diet: Sepiolite, 6.043 g; pig vitamin, 750 mg; Fe, 150 mg; Cu, 150 mg; Mn, 80 mg; Zn, 120 mg; Se, 0.3 mg; Co, 1 mg; I, 0.3 mg; VB4 1000 mg.

### Real-Time Quantitative RT-PCR

Total RNA was isolated from liquid nitrogen–frozen kidney, liver, and spleen using TRIZOL reagent (Invitrogen, USA) and then treated with DNase I (Invitrogen, USA) according to the instructions of the manufacturer. Synthesis of the first strand (cDNA) was performed with PrimeScript Enzyme Mix 1, RT Primer Mix, and 5 × PrimerScript Buffer 2. The reverse transcription was conducted at 37 °C for 15 m, 85 °C for 5 s. Primers (Table 3) used in this study were presented in the previous study^23–25^. β-actin was used as a housekeeping gene to normalize target gene transcript levels. Real-time PCR was performed according to our previous study^3^. Briefly, 1 μl cDNA template was added to a total volume of 10 μl containing 5 μl SYBR Green mix, 0.2 μl Rox, 3 μl ddH2O, and 0.4 μl each of forward and reverse primers. We used the following protocol: (i) pre-denaturation programma (30 s at 95 °C); (ii) an amplification and quantification program consisting of repeated 40 cycles (5 s at 95 °C and 30 s at 60 °C); (iii) a melting curve program (extention at 72 °C). Relative expression was expressed as a ratio of the target gene to the control gene using the formula 2^−(ΔΔCt)^, where ΔΔCt = (Ct_Target_ − Ct_β-actin_) _treatment_ − (Ct_Target_ − Ct_β-actin_) _control_^26^. Relative expression was normalized and expressed relative to the expression in the control group.

**Table 3:** Primers used for quantitative reverse transcription PCR.

Gene	Accession no.	Sequence(5′-3′)
β-actin	XM_0031242803	F: CTGCGGCATCCACGAAACT R: AGGGCCGTGATCTCCTTCTG
TLR3	NM_001097444.1	F: GCAAGAACTCACAGGACAGGAA R: GGCGAAAGAGTCGGTAGTCAA
TLR4	NM_001113039.1	F: CCGTCATTAGTGCGTCAGTTCT R: TTGCAGCCCACAAAAAGCA
TLR7	NM_001097434.1	F: TTGTTCCATGTATGGGCAGA R: TTCCAGGTTGCGTAGCTCTT
TLR8	NM_214187.1	F: TTCCCACATCCCAGACTTTC R: TTGCTTTGGTTGATGCTCTG
TLR9	NM_213958.1	F:AGCCTCAACCTGTCCTTCAATTACC R: CTGAGCGAGCGGAAGAAGATGC
IL-1β	NM_214055.1	F:GCTAACTACGGTGACAACAA R:TCTTCATCGGCTTCTCCACT
IL-4	NM_214340.1	F:CCCGAGTGTCAAGTGGCTTA R:TGATGATGCCGAAATAGCAG
IL-6	NM_214403.1	F:TCCAGCATCATTGCATCATC R:GGCTCCACTCACTCCACAAG
IL-8	NM_213867.1	F:TGAGAAGCAACAACAACAGCA R:CAGCACAGGAATGAGGCATA
IL-10	NM_214041.1	F:GGGCTATTTGTCCTGACTGC R:GGGCTCCCTAGTTTCTCTTCC
IL-12	NM_214097.2	F:ATCTCGGTTGGTGTTGTTCC R:GGGTATCTCGTCCTCTGTCC
IFN-γ	NM_2139481	F:TTCAGCTTTGCGTGACTTTG R:GGTCCACCATTAGGTACATATG
TNF-α	NM_214022.1	F:ACAGGCCAGCTCCCTCTTAT R:CCTCGCCCTCCTGAATAAAT
TGF-β1	NM_214015.1	F:AAGCGGCAACCAAATCTATG R:CCCGAGAGAGCAATACAGGT
Myd88	NM_001099923	F:GTGCCGTCGGATGGTAGTG R:TCTGGAAGTCACATTCCTTGCTT

### Western blot analysis

The expression of protein in the kidney, liver and spleen was determined by Western blot analysis as described previously. Briefly, about 50 µg of total protein obtained from samples were extracted by a reducing SDS-PAGE electrophoresis. The proteins were transferred onto polyvinylidene difluoride membranes and blocked with 5% nonfat milk in tris-Tween-buffered saline buffer (20 mM tris, pH 7.5, 150 mM NaCl, and 0.1% Tween 20) for 1.5 hour. Then the primary antibodies were incubated overnight at 4°C and the horseradish peroxidase-conjugated secondary antibodies were subsequently incubated for 1.5 hour at room temperature before development of the blot using the Alpha Imager 2200 software (Alpha Innotech Corporation, CA, USA). We quantified the resultant signals and normalize the data to the abundance of β-actin according to our previous reports.

### Statistical analysis

All data were analyzed between two groups using the student’s T test (SPSS 16.0 software). Data are expressed as the mean ± SEN. Differences of p < 0.05 are considered significant.
